# Construction of a full-length infectious bacterial artificial chromosome clone of duck enteritis virus vaccine strain

**DOI:** 10.1186/1743-422X-10-328

**Published:** 2013-11-06

**Authors:** Liu Chen, Bin Yu, Jionggang Hua, Weicheng Ye, Zheng Ni, Tao Yun, Xiaohui Deng, Cun Zhang

**Affiliations:** 1 Institute of Animal Husbandry and Veterinary Medicine, Zhejiang Academy of Agricultural Sciences, Hangzhou 310021, China

**Keywords:** Duck enteritis virus, Bacterial artificial chromosome, Infectious clone

## Abstract

**Background:**

*Duck enteritis virus* (DEV) is the causative agent of duck viral enteritis, which causes an acute, contagious and lethal disease of many species of waterfowl within the order *Anseriformes*. In recent years, two laboratories have reported on the successful construction of DEV infectious clones in viral vectors to express exogenous genes. The clones obtained were either created with deletion of viral genes and based on highly virulent strains or were constructed using a traditional overlapping fosmid DNA system. Here, we report the construction of a full-length infectious clone of DEV vaccine strain that was cloned into a bacterial artificial chromosome (BAC).

**Methods:**

A mini-F vector as a BAC that allows the maintenance of large circular DNA in *E. coli* was introduced into the intergenic region between UL15B and UL18 of a DEV vaccine strain by homologous recombination in chicken embryoblasts (CEFs). Then, the full-length DEV clone pDEV-vac was obtained by electroporating circular viral replication intermediates containing the mini-F sequence into *E. coli* DH10B and identified by enzyme digestion and sequencing. The infectivity of the pDEV-vac was validated by DEV reconstitution from CEFs transfected with pDEV-vac. The reconstructed virus without mini-F vector sequence was also rescued by co-transfecting the Cre recombinase expression plasmid pCAGGS-NLS/Cre and pDEV-vac into CEF cultures. Finally, the *in vitro* growth properties and immunoprotection capacity in ducks of the reconstructed viruses were also determined and compared with the parental virus.

**Results:**

The full genome of the DEV vaccine strain was successfully cloned into the BAC, and this BAC clone was infectious. The *in vitro* growth properties of these reconstructions were very similar to parental DEV, and ducks immunized with these viruses acquired protection against virulent DEV challenge.

**Conclusions:**

DEV vaccine virus was cloned as an infectious bacterial artificial chromosome maintaining full-length genome without any deletions or destruction of the viral coding sequence, and the viruses rescued from the DEV-BAC clone exhibited wild-type phenotypes both *in vitro* and *in vivo*. The generated infectious clone will greatly facilitate studies on the individual genes of DEV and applications in gene deletion or live vector vaccines.

## Background

Duck virus enteritis (DVE), also known as duck plague, is an acute contagious infection of ducks, Muscovy ducks, geese, and swans (order *Anseriformes*) that is caused by *duck enteritis virus* (DEV). According to the most recent virus taxonomy reported in 2012 by the International Committee on Taxonomy of Viruses (ICTV), DEV (also referred to as *Anatid herpesvirus 1*) is classified into the genus *Mardivirus* and the subfamily *Alphaherpesvirinae* of *Herpesviridae*[1]. This virus can infect ducks of both sexes of a wide age and has high morbidity and mortality. The disease is characterized by extensive hemorrhaging and necrosis, most commonly in the digestive and lymphoid organs. DVE has resulted in significant economic losses in domestic and wild waterfowls worldwide. At present, the major approaches to preventing and controlling lethal DEV infections in ducks is to inoculate ducks with live attenuated DEV vaccines.

Herpesvirus is a large, enveloped virus with four structural components, including linear double-stranded DNA, an icosahedral capsid, an amorphous tegument, and a bilayer lipid envelope. The genomes of these viruses differ in size, sequence arrangements, and base composition, and they also vary significantly with respect to the presence and arrangement of inverted and directly repeated sequences [2,3]. Partial or complete genomic sequences of DEV have been obtained, and there has been discrepancies found between these sequences, which demonstrate that although similar to other herpesviruses, the DEV genome also varies [4-10]. In general, the DEV genome is approximately 158 kb and contains 78 ORFs predicted to encode potential functional proteins. In addition, the genome also contains a unique long (U_L_) region, a unique short (U_S_) region, a unique short internal repeat (IR) region, and a unique short terminal repeat (TR) region.

Bacterial artificial chromosomes (BACs) have proven to be useful vectors for cloning large gene fragments, including viral genomes. A combination of a viral BAC system and various *Escherichia coli*-based recombination systems is a highly reliable and efficient method for generating a variety of different modifications of BAC clones, which are fundamental tools for applications as diverse as the elucidation of viral gene or domain function, the generation of transgenic animals, and the construction of gene therapy or vaccine vectors [11-13]. The genomes of many herpesviruses, including cytomegalovirus (CMV), Marek’s disease virus (MDV), koi herpesvirus (KHV), varicella zoster virus (VZV), bovine herpesvirus type 1 (BoHV-1), equine herpesvirus 4 (EHV-4), and pseudorabies virus (PRV), have been cloned as bacterial artificial chromosomes in *E. coli*[14-20]. The infectious viral BAC of DEV virulent strain 2085 was first constructed in 2011 by Wang *et al*. [21]. Then, Liu *et al*. established a system to generate DEV vaccine strain by transfecting overlapping fosmid DNAs [22]. Here, we report the construction of an infectious clone of a Chinese DEV vaccine C-KCE strain that has been commercialized and widely used in preventing DEV infection in China. This DEV BAC system will facilitate the studying of DEV biology and gene functions as well as novel vaccines.

## Results

### Generation of recombinant DEV harboring mini-F vector sequences

**Figure 1:**
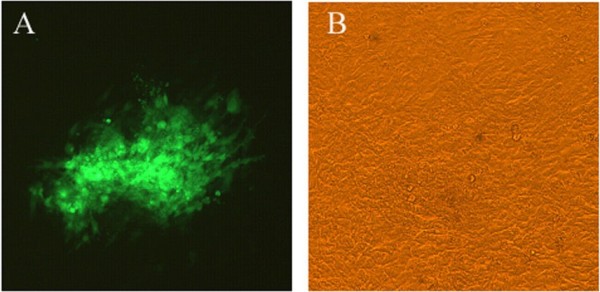
**Homogeneous population of rDEV in CEFs (100×). A**. Fluorescence under UV excitation; **B**. Phase contrast.

CEFs were transfected with the linearized transfer vector pHA2-UL18-UL15 and then infected with DEV vaccine strain (DEV-vac). Drug screening, combining plaque picking and passaging of end-point dilutions of recombinant progeny virus identified by GFP expression, were performed. Purified recombinant rDEV, which carries mini-F sequences inserted within the non-encoding region between UL18 and UL15B, was obtained after 10 rounds of plaque and end-point purification (Figure 1).

### Generation and validation of a supercoiled DEV-vac-BAC clone

**Figure 2:**
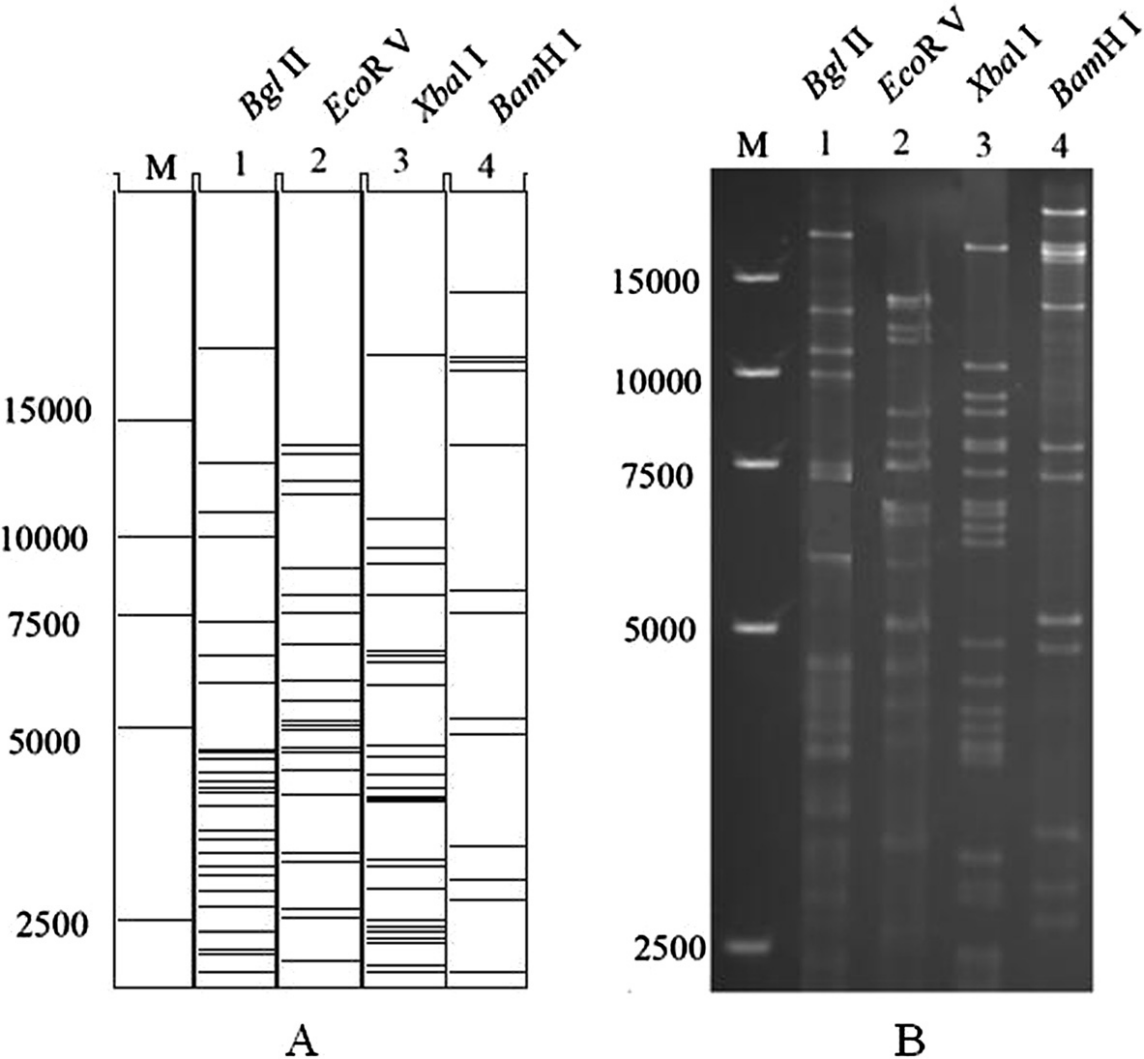
**Restriction fragment analysis of full-length BAC clone pDEV-vac. A**. The patterns corresponded exactly to the predictions based on the complete DEV genome (KF487736), as shown by *in silico* digests using Vector NTI. **B**. BAC DNA was isolated and digested with four different enzymes (*EcoR* V, *Bgl* II, *Bam*H I, and *Xbal* I) and separated with a 1.0% agarose gel. The sizes of a molecular weight marker (15,000-bp marker, Takara) are given.

Circular viral DNA was isolated from rDEV-infected cells by the SDS-proteinase K method and transferred into *E. coli* by electroporation. Several chloramphenicol-resistant colonies were obtained 36 h after plating of electroporated cells, and RFLPs (restriction fragment length polymorphisms) were determined to confirm that a full-length DEV-vac BAC clone was indeed generated. The DNA of two selected colonies was isolated and digested with four enzymes: *Eco*R V, *Bgl* II, *Bam*H I, and *Xba*l I. The electrophoresis results indicated that the bands from two colonies were completely identical. When the RFLP patterns were compared to *in silico* predictions, which were based on the reference whole genome sequence of the DEV vaccine strain [GenBank: KF487736] [10], the obtained *Bam*H I pattern matched those of the predictions well, and the other enzyme digestion patterns displayed a few differences from the predictions (Figure 2).

### Nucleotide sequence of pDEV-vac

To further evaluate the integrity of the generated infectious clones, whole genome sequencing of pDEV-vac [GenBank: KF693236] and comparison with the reference sequence [GenBank: KF487736] was performed. The results of the whole genome sequencing of pDEV-vac revealed that there were only four nucleotide changes in the viral coding sequence: a silent mutation in UL36, an H_1114_ P mutation in UL36, an I_205_V mutation in UL23, and an R_185_Q mutation in UL3. There were seven site changes, including two 80-nt repetitive sequence deletions in the non-coding region, compared with the published sequence (Table 1).

**Table 1:** List of nucleotide sequence different between cloned pDEV-vac [GenBank: KF693236] and the published DEV vaccine sequence [GenBank: KF487736]

**A. Changes in coding sequences**		
**Gene (according to KF487736)**	**Modification (nt position)**	**Effect**
UL36	A to G (41 128)	Silent
UL36	A to C (43 596)	H to P
UL23	A to G (75 096)	I to V
UL3	C to T (110 795)	R to Q
**B. Changes in non-coding sequences**		
**Modification (nt position)**		**Comment**
T to C (59 643)		Between UL30 and UL29
Deletion 80 nt : GGGTTCCAAAGGTTTTACGGTA		
TTAGGGTTAGGGTTGGCAGGGTTCCAAAGGTTT		
TACGGTATTAGGGTTAGGGTTGGCA (119 773–119 852)		Between LORF2 and IR
Insertion: AA (129 421–129 422)		Between IR and U_S_1
T to G (143 005)		Between U_S_7 and U_S_8
Insertion: TT (147 986–147 987)		Between U_S_1 and TR
Deletion 80 nt: CCTAATACCGTAAAACCTTTGGAACC		
CTGCCAACCCTAACCCTAATACCGTAAAACCTTTGG		
AACCCTGCCAACCCTAAC (157 498–157 877)		Between TR and LORF11
Insertion: G (158 090)		Between TR and LORF11

To construct an infectious DEV clone using a BAC system, the process, which successively includes “virus passage and recombination in cells”, “from recombinant virus to BAC” (that is, from cells to bacteria), and “from BAC to virus” (that is, from bacteria back to cells) can potentially lead to changes in the viral genome. To further illuminate in which step these mutations were introduced, we sequenced 11 sites in four virus (DEV-vac, rDEV, rDEV-BAC, and rDEV-Cre) genomes and compared them with pDEV-vac and the reference sequence [GenBank: KF487736]. The results are shown in Table 2. The results indicated that the majority of changes occurred in the “virus passage and recombination in cells” step, including A_41128_G, A_43596_C, A_75096_G, and C_110795_T_._ A minor mutation occurred in the “from cells to bacteria” step, e.g., an 80-nt deletion in 119773–119852. Some changes occurred in both the “virus passage and recombination in cells” and “from cells to bacteria” steps, including changes in sites 129421–129422, 147986–147987, and 157498–157877. In addition, no mutations occurred in the “from bacteria back to cells” step. T_59643_, T_143005_ and G_158090_ were the same as in the parental virus DEV-vac.

**Table 2:** Comparasion of mutated sites in pDEV-vac (KF693236), the published DEV vaccine sequence (KF487736)), and four viruses DEV-vac, rDEV, rDEV-BAC and rDEV-Cre

**Modification (nt position, according to KF487736)**	**pDEV-vac (GenBank: KF693236)**	**Reference strain (GenBank: KF487736)**	**DEV-vac**	**rDEV**	**rDEV-BAC**	**rDEV-Cre**
41 128	A	G	G	A	A	A
43 596	A	C	C	A	A	A
75 096	A	G	G	A	A	A
110 795	C	T	T	C	C	C
59 643	T	C	T	T	T	T
	Deletion 80 NT:	GGGTTCCAAAGG	GGGTTCCAAAGG	GGGTTCCAAAGG	Deletion 80 NT:	Deletion 80 NT:
	— — — — — — — —	TTTTACGGTATTA	TTTTACGGTATT	TTTTACGGTATT	— — — — — — — —	— — — — — — — —
	— — — — — — — —	GGGTTAGGGTTG	AGGGTTAGGGTT	AGGGTTAGGGTT	— — — — — — — —	— — — — — — — —
119 773–119 852	— — — — — — — —	GCAGGGTTCCAA	GGCAGGGTTCCA	GGCAGGGTTCCA	— — — — — — — —	— — — — — — — —
	— — —	AGGTTTTACGGT	AAGGTTTTACGG	AAGGTTTTACGG	— — —	— — —
		ATTAGGGTTAGG	TATTAGGGTTAG	TATTAGGGTTAG		
		GTTGGCA	GGTTGGCA	GGTTGGCA		
129 421–129 422	Insertion: AA	–	–	A-	AA	AA
143 005	T	G	T	T	T	T
147 986–147 987	Insertion: TT	–	–	T-	TT	TT
	Deletion 80 nt:	CCTAATACCGTA	CCTAATACCGTA	Deletion 40 nt:	Deletion 80 nt:	Deletion 80 nt:
		AAACCTTTGGAA	AAACCTTTGGAA			
	— — — — — — — —	CCCTGCCAACCC	CCCTGCCAACCC	CCTAATACCGTA	— — — — — — — —	— — — – – – –
				AAACCTTTGGAA	— — — — — — — —	— — — – – – –
				CCCTGCCAACCC	— — — – – – –	— — — – – – –
				TAAC — — — — — — — — —	— — —	— — —
157 498–157 877	— — — — — — — —	TAACCCTAATAC	TAACCCTAATAC			
	— — — — — — — —	CGTAAAACCTTT	CGTAAAACCTTT			
	— — —	GGAACCCTGCCA	GGAACCCTGCCA	— — — — — — — —		
		ACCCTAAC	ACCCTAAC			
158 090	Insertion : G	-	G	G	G	G

### Reconstitution and characterization of BAC-derived DEV

pDEV-vac DNA was transfected into CEFs by calcium phosphate precipitation to produce the recombinant virus rDEV-BAC. A typical cytopathic effect (CPE) with green fluorescence was observed in the cell monolayer 4 days later. To exclude the effect of additional BAC sequences on the virus, the BAC sequence, including the *xgpt* and *gfp* genes, was excised by site-specific recombination using flanking loxP sites. This excision was achieved by co-transfecting the Cre recombinase expression plasmid pCAGGS-NLS/Cre and pDEV-vac into the CEF cultures. Following this step, a typical DEV CPE without green fluorescence appeared; then, the cloned virus without the BAC sequence, named rDEV-Cre, was obtained by limiting dilution and plaque purification. The correct excision of BAC sequences from the recombinant virus rDEV-Cre was confirmed by PCR and sequencing (data not shown).

**Figure 3:**
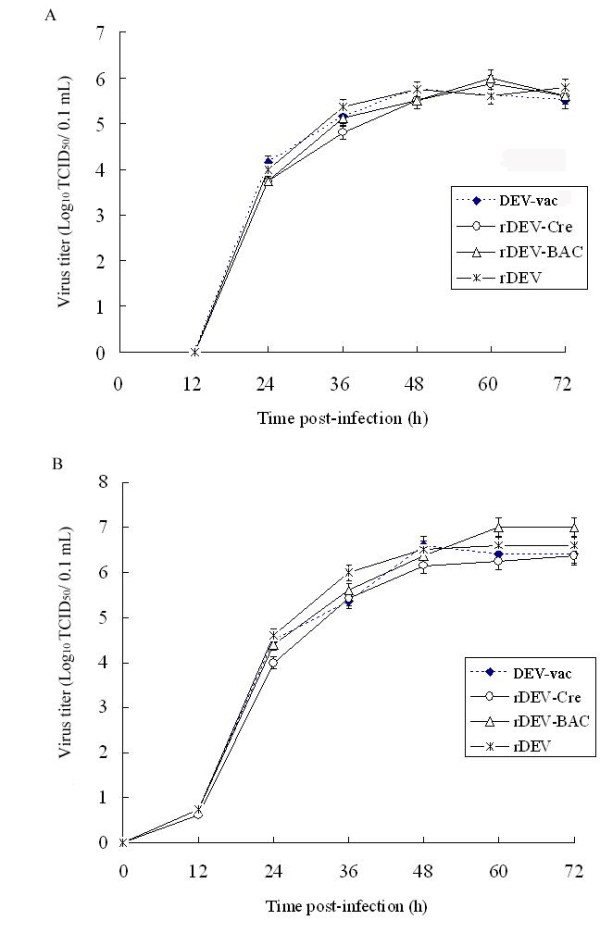
**Multi-step growth curves of rDEV, rDEV-BAC, rDEV-Cre, and DEV-vac.** Comparison of the *in vitro* growth of viruses reconstructed with parental DEV. The virus titers of infected-cells **(A)** and supernatants **(B)** were determined at different times (0, 12, 24, 36, 48, 60, and 72 h) after inoculation of approximately 0.02 MOI of cell-free viruses of rDEV, rDEV-BAC, rDEV-Cre, and DEV-vac. The multi-step growth curves were computed from three independent experiments.

**Figure 4:**
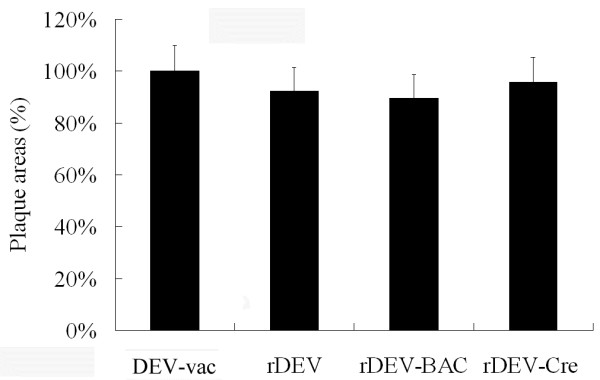
**Plaque area measurement of DEV-vac, rDEV, rDEV-BAC, and rDEV-Cre on CEFs.** The means and standard deviations of sizes of 100 plaques of each virus were measured with Image J software. The mean of the plaque area of DEV-vac was set at 100%. Standard deviations are shown with the error bars.

To compare the growth of the recombinant virus and the parental virus, the multi-step growth kinetics of the reconstructed viruses were determined and compared with DEV-vac. As shown in Figure 3, all reconstituted viruses, rDEV, rDEV-BAC, and rDEV-Cre (both intracellular and extracellular), exhibited growth characteristics that were virtually identical to each other and to those of parental DEV-vac. In addition, the virus titers steadily increased from 12 to 60 h post-infection. When the plaque areas of the reconstructed viruses rDEV, rDEV-BAC, and rDEV-Cre were separately compared to parental virus DEV-vac, it was discovered that the plaque areas were approximately 7.63%, 10.38%, and 4.16% smaller, respectively, than those formed by DEV-vac when measured on day 4 p.i., but there were no significant differences between the groups (*P* = 0.241; *P* = 0.05; *P* = 0.485), and there was also no significant difference between rDEV and rDEV-BAC (*P =* 0.554) (Figure 4). These data demonstrate that the insertion of mini-F sequence into DEV’s genome and/or proliferation of viral genome in *E. coli* had a slight effect on the viral plaque area.

### Reconstructed virus could protect ducks against virulent DEV challenge

**Figure 5:**
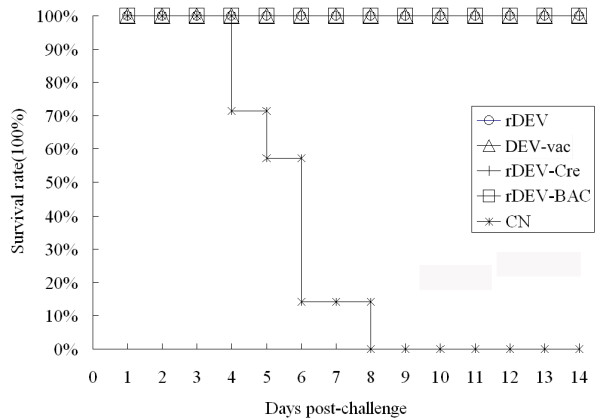
**Protective efficacy of the reconstructed viruses against lethal DEV challenge.** Groups of 7 ducks were vaccinated intramuscularly with 1 × 10^5^ TCID_50_ of DEV-vac, rDEV, rDEV-BAC, rDEV-Cre, or with culture medium as a control, and 2 weeks later, all groups were challenged with lethal DEV. The ducks were monitored daily for 2 weeks after challenge.

To investigate whether the reconstructed viruses could protect ducks against virulent DEV challenge, the ducks were first inoculated with 1 × 10^5^ TCID_50_ of DEV-vac, rDEV, rDEV-BAC, rDEV-Cre, or with culture medium as a control for 2 weeks and then challenged with highly virulent DEV. Four days later, the clinical symptoms of duck viral enteritis and death of the ducks were observed only in the negative control groups, and all ducks in the control groups died within 8 days post-challenge. The other groups, which had been previously inoculated with reconstructed viruses or DEV-vac, remained healthy during the 2-week observation period. The survival rates of the different groups are summarized in Figure 5.

Tissues from all birds were also examined for pathological lesions. The gross lesions were characterized by vascular damage, with tissue hemorrhages, especially in liver, spleen, esophagus, and cloaca, and there was annular band necrosis and hemorrhage in the intestinal tract, along with diphtheroid lesions in the mucosal surfaces of the esophagus and cloaca.

## Discussion

Although DEV is an important pathogen in waterfowl that causes high morbidity and mortality in infected birds, little is known about the function of viral genes and proteins, the molecular and cellular mechanisms of different phases of the DEV life cycle, and the mechanisms involved in DEV virulence and pathogenicity. Full-length genomic clones from DEV strains allowing the recovery of infectious virus would be an invaluable tool for various in-depth investigations on these questions. To date, there have been only two reports detailing the construction of DEV infectious clones, and based on the established platform, exogenous genes such as the H5N1 HA gene were successfully expressed. In one case, the recombinant virus could provide fast and complete protection against H5N1 avian influenza virus infection in ducks [21,22]. These results demonstrate that DEV is a potential viral vector.

Compared with an overlapping fosmid DNA system, there are more advantages to cloning the viral genome into a BAC system, as it allows stable maintenance the viral genome in *E. coli* and also allows a variety of different modifications to the viral genome to be easily generated using various *E. coli*-based recombination systems. It has been reported that the insertion of foreign genes into the intergenic region between genes UL15 and UL18 in BoHV was stable and had no effect on viral growth in cell culture [23]. In this study, the same site was chosen for construction of infectious viral BAC for the DEV vaccine strain. This infectious DEV clone was successfully constructed by inserting the bacterial mini-F plasmid sequences flanked by loxP sites into the intergenic region between genes UL15B and UL18 of DEV without deletion of any viral sequence through homologous recombination. Our results demonstrate that exogenous sequence insertion between the UL15B and UL18 genes had no effect on DEV replication. The plaque area measurement and multi-step growth kinetics results reveal that insertion of mini-F sequence into DEV’s genome and/or proliferation of the viral genome in *E. coli* had a slight effect on virus transmission during cell-to-cell spread but did not affect the virus growth pattern *in vitro*. Furthermore, these processes did not increase the virulence of the DEV-vaccine strain in ducks and had no effect on its ability to generate immunoprotection to lethal DEV challenge.

A process involving procedures “from cells to bacteria and again back to cells” is experienced in constructing the infectious DEV clone with the BAC system that could potentially result in some changes to the viral genome. To select a clone and/or virus that was closest to the parental strain in terms of sequence, a series of studies, including restriction enzyme digestion and genome sequencing of the viral BAC clones, determination of rescued virus plaque sizes and growth kinetics *in vitro*, and tests of immunoprotection capability in ducks, were conducted. Although there were a few mismatches and changes in the viral genome in pDEV-vac compared with the reference sequence, the obtained reconstructed rDEV-Cre had almost identical immunoprotection characteristics to the parental virus. Whether these mismatches and changed sites have an effect on the virus in other areas of characterization remains to be explored.

Two routine methods have been adopted to construct recombinant virus containing a BAC sequence. One is to co-transfect the transfer vector and viral genome into cells (co-transfect method). The other is to transfect the transfer vector into cells first and to then follow with a virus infection (transfection-infection method). For cells with low transfection efficiency, the latter is more efficient. Primary chicken embryo fibroblasts are difficult to transfect, and low transfection efficiency limits homologous recombination of duck enteritis virus genome to transfer plasmids. In this study, we adapted the transfection of suspended CEFs using the calcium phosphate method to improve transfection efficiency.

In animal experiments, viral DNA was detected using PCR of serum samples of all ducks immunized for 2 weeks without challenge with highly virulent DEV. The results indicated that viral DNA still existed in the blood of ducks in the immunized groups (data not shown). This finding demonstrates that those viruses were still active in ducks after being injected into the body for 2 weeks, and that viruses lies in body for a long time may be the prerequisite for live attenuated vaccines to express viral antigens that periodically stimulate the immune system to establish long-term immunoprotection.

## Conclusions

We established an infectious bacterial artificial chromosome clone of a widely used attenuated live vaccine strain in China. The results revealed that the rescued virus has similar replication and immunogenic characteristics as its parental strain. Based on this infectious clone, it would be possible to develop novel vaccines, including gene-deletion vaccines, or other marker vaccines for serological differentiation of DEV naturally infected from vaccinated animals. In addition, it would also be possible to use a viral vector to construct a bivalent or multivalent vaccine.

## Materials and methods

All research was approved by the relevant committees at the Zhejiang Academy of Agriculture Sciences.

### Virus strain and cells

The DEV vaccine virus (C-KCE strain, a commercial DEV vaccine in China, CVCC AV1221) and the standard highly virulent CHv strain (CVCC AV1221) used in this study were provided by the China Institute of Veterinary Drugs Control (Beijing, China) [24]. Chicken embryo fibroblasts (CEFs) were prepared from 10-day-old specific-pathogen-free (SPF) embryonated eggs (Zhejiang JianLiang Bioengineering Co., Ltd., Hangzhou, China) according to standard procedures and cultured in DMEM (Gibco-BRL) supplemented with 8% FBS, 100 U of penicillin/mL and 100 μg streptomycin/mL.

### Construction of transfer vector for homologous recombination

**Figure 6:**
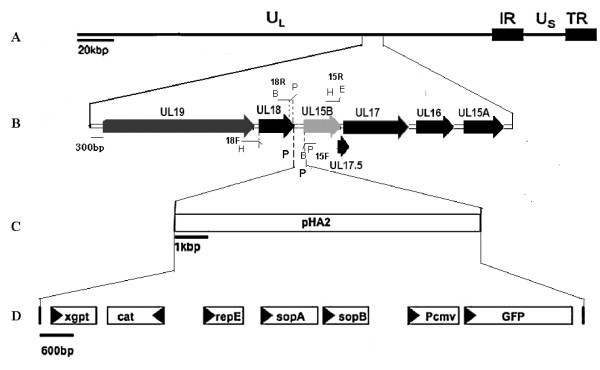
**Schematic drawing of the cloning procedure to introduce the pHA2 vector into the DEV-vac genome. (A)** Diagram of the 158 kb DEV-vac genome. Abbreviations: U_L_, unique long region; U_S_, unique short region; TR, terminal repeat; IR, internal repeat. **(B)** The region of interest from UL19 to UL15A genes in DEV-vac genome. **(C)** The HA2 segment was inserted between UL15B and UL18 by transfection of transfer plasmid following infection with the DEV-vac genome. **(D)** The locations of the genes in pHA2 (including mini-F plasmid) are depicted by boxes. *xgpt*, xanthine-guanine phosphoribosyltransferase; cat, chloramphenicol resistance gene; *gfp*, green fluorescent protein gene; rep E, replication gene E; sopA, sopB, sopC, partitioning genes A, B, C; Pcmv, cytomegalovirus promoter. B, *Bam*H I; P, *Pac* I; H, *Hin*d III; E, *Eco*R I.

Two pairs of primers, 18 F/18R and 15 F/15R (Table 3), were designed to amplify the UL18 and UL15B fragments, respectively, of the DEV-vac strain. The PCR products were 1083 nt and 1258 nt in size, respectively. The fragments were cloned into a pMD18-T vector (Takara) using enzyme sites present in the primers, and the resulting plasmid was designated pMD-UL15-UL18. Then, a mini-F vector pHA2 containing *xgpt* and *gfp* genes was released as a *Pac* I fragment from plasmid pDS-pHAII, which was a gift from Dr. M. Messerle [25], and cloned into the *Pac* I site present in pMD-UL15-UL18 to construct the transfer vector pHA2-UL18-UL15 (Figure 6).

**Table 3:** Primers used in this study

**Primer**	**Sequence**	**Domain introduced**
18 F	5’-AAAGCTTCGACGAGAGTATCAGCACTCATC-3’	*Hin*d III
18R	5’-AGGATCC*TTAATTAA*CAAGACAGACAAGTATTGCTTGG-3’	*Bam*H I-*Pac* I
15 F	5’-AGGATCC*TTAATTAA*GACATTTCCAAGCAATACTTGTCTGTC-3’	*Bam*H I-*Pac*I
15R	5’-AGAATTC*AAGCTT*ATCGACGAACAACTAAGAGTTTGC-3’	*EcoR* I-*Hin*d III
UL18-s	5’- AAGGACCGCCTACTATCAAA-3’	
gfp-s	5’- AGAAGAACGGCATCAAGGTG-3’	
xgpt-s	5’- TCAGTCGGCTTGCGAGTTTA-3’	
UL15-s	5’-AAACGGGTGGCTGTGCTGAT-3’	

Note. Restriction enzyme recognition sequences are underlined and italicized.

### Construction of the BAC clone of DEV-vac

Construction of the BAC clone of the DEV-vac strain was conducted by inserting the bacterial mini-F plasmid sequences flanked by loxP sites into the intergenic region between genes UL15B and UL18 of DEV through homologous recombination (Figure 6).

(1) Transfection of DNA into suspended CEF cells and virus infection

The transfer vector pHA2-UL18-UL15 was prepared according to the protocol of the Qiagen® plasmid purification handbook (Qiagen) and then digested with *Hin*d III and precipitated with 1/10 volume of 3 M sodium acetate and the DNA was dissolved in TE buffer at a concentration of 0.5 μg/μL. A calcium phosphate method was employed to transfect suspended cells according to Morgan *et al.*[26] with modifications. In detail, primary chicken embryo fibroblasts (CEFs) from 10-day-old SPF chicken embryos were cultured in flasks for 16 to 24 h. Once the cells had formed a confluent monolayer, the cells were digested by trypsin-EDTA and seeded in a 6-well plates with a density of 2.4 × 10^6^ cells/well in growth medium (DMEM containing 8% fetal calf serum) without antibiotics. Transfection was operated according to ProFection Mammalian Transfection System - Calsium Phosphate (Promega, USA) protocol. 2 μg of linearized transfer plasmid was transfected into suspended CEFs per well. Following a 4-h incubation at 37°C, the cultures were treated for 3 minutes with 15% glycerol in 1 × HBSP (1.5 mL/well). Subsequently, a series of doses of DEV-vac virus stock (200 μL, 100 μL, 50 μL, 25 μL,.) were added to each well, and after 2 h of incubation at 37°C, the culture medium was replaced with growth medium without antibiotics.

(2) Selection of DEV recombinants expressing the *xgpt* gene

The DEV-vac-infected cells were collected when 80% of the cells displayed CPE. Then, the collected cells were added to one well of the 6-well-plate cells previously incubated with selection medium containing 300 μg/mL MPA (mycophenolic acid) (Sigma), 60 μg/mL xanthine, 100 μg/mL hypoxanthine, 2% FBS, 100 U of penicillin/mL, and 100 μg streptomycin/mL The selection medium was replaced at 1-d intervals. GFP-positive plaques were isolated and transferred to newly prepared CEFs. Following 10 cycles of plaque picking, the purified recombinant virus rDEV with Xgpt and GFP labels was obtained.

(3) Generation and validation of a supercoiled DEV-vac-BAC clone

To generate the circular, supercoiled form of the BAC clone, the cells infected with the recombinant virus in the early phase (approximately 30-50% CPE) were collected, and the total DNA was extracted from the cells using the SDS-proteinase K method as described previously [24]. Then, 1 μg of DNA was electroporated into *E. coli* DH10B competent cells (Invitrogen) according to the manufacturer’s instructions. The transformed bacteria were incubated in 1 mL of SOC medium for 1 h and then plated on LB agar containing 30 μg/mL chloramphenicol. The next day, single colonies were picked and cultured in liquid LB medium, and BAC DNAs were isolated by alkaline lysis of *E. coli*. Then, BAC DNAs of pDEV-vac were identified by RFLPs, and the infectivity of the pDEV-vac was examined by transfecting the BAC DNA into CEF cultures.

### Reconstitution and characterization of BAC-derived DEV

Next, 4 μg of pDEV-vac DNA was transfected into CEFs by calcium phosphate precipitation as mentioned above. The cells were then cultured with DMEM supplemented with 8% FBS for 3–6 days. The virus collected was named rDEV-BAC. The mini-F sequence in the rDEV-BAC genome was excised by co-transfecting 1 μg each of the Cre recombinase expression plasmid pCAGGS-NLS/Cre (kindly provided by Dr. N. Osterrieder, Freie Universität Berlin, Berlin, Germany) and pDEV-vac into the CEF cultures. The virus rDEV-Cre without mini-F vector sequences was obtained from the transfected CEF cultures by limiting dilution and plaque purification and then identified by PCR amplification with the primer pairs 18 F/15R (Table 3).

Multi-step growth kinetics of those reconstructed viruses were determined and compared with the parental virus DEV-vac. Briefly, the CEFs were infected with approximately 0.02 MOI of cell-free viruses of rDEV, rDEV-BAC, rDEV-Cre, and DEV-vac. The cells and culture supernatant were harvested at different times (0, 12, 24, 36, 48, 60, and 72 h) after virus infection. The cells were collected by trypsin digestion following two washes with phosphate-buffered saline (PBS, pH 7.0) and were then suspended with 1 mL of DMEM-2% FBS and an equal volume of supernatant. The collected cells and supernatants were stored at -70°C until all samples had been collected. Before titration on CEFs, the cells were treated with a Tissuelyser-24 at 65 Hz for 60 s and centrifuged at 4000 rpm for 5 min, and then 100 μL of lysis supernatant and culture supernatant were taken for titering via the TCID_50_ test according to standard virological methods. The multi-step growth curves were computed from three independent experiments.

The plaque sizes of rDEV, rDEV-BAC, rDEV-Cre, and DEV-vac were also measured. The viruses were serially diluted and plated onto CEFs seeded in a 12-well plate, and 2 h later, the culture medium was replaced with DMEM containing 1.5% methylcellulose. After a 4-day-incubation at 37°C, the cells were treated with 5% formaldehyde solution and stained with 1% crystal violet/70% ethanol. Then, for every virus, 100 plaques were randomly selected and their size measured using Image J software (http://rsb.info.nih.gov/ij/). Statistical analyses to compare the differences in the plaque sizes between the four strains were conducted using one-way ANOVA with SPSS 11.5 software.

### Sequencing the BAC with Ion torrent technology

The sequence of pDEV-vac was determined with Ion Torrent technology from the Invitrogen company. Briefly, a DNA fragment library was first prepared with the Ion Plus Fragment Library Kit (Life Technologies). The resulting DNA library was then bound to ionic sphere particles (ISPs) by specialized adaptors ligated onto the ends of the fragmented DNA. Then, the DNA templates on ISPs were amplified using PCR with primers specific for the adaptors. Finally, sequencing was performed using an Ion Torrent Personal Genome Machine (PGM) (Life Technologies) platform, and the data were assembled using Torrent Suite software (Life Technologies). The obtained sequence was aligned with several published whole genome sequences of DEV vaccine strains [GenBank: EU082088.2, GenBank: KF487736, and GenBank: NC_013036.1] with Vector NT1 Advance 9 software (Invitrogen). The regions of the sequenced pDEV-vac that were different from several reference nucleotide sequences were amplified by PCR and sequenced again using standard chain termination protocols. The ultimate sites of difference between pDEV-vac and the reference strain KF487736 were determined, and the corresponding sites in four viral (DEV-vac, rDEV, rDEV-BAC, and rDEV-Cre) genomes were also determined by PCR and sequenced.

### Vaccine efficacy in ducks

A total of 35 20-day-old specific-pathogen-free (SPF) ducks (Harbin Veterinary Research Institute, CAAS, China) were used for these studies. The animals were randomly assigned to five groups (*n* = 7) and housed in separate rooms. Four groups were separately inoculated intramuscularly (i.m.) with 0.5 mL of medium containing 1 × 10^5^ TCID_50_ (50% tissue culture infection dose) of rDEV, rDEV-BAC, rDEV-Cre, and DEV-vac. The fifth group served as a negative control and was inoculated with medium without virus. Two weeks later, all groups were challenged with a 100-fold 50% duck lethal dose (DLD_50_) of a highly virulent DEV CHv strain i.m. Then, the signs of disease and death were observed within 2 weeks.

## Abbreviations

DEV: Duck enteritis virus; CEFs: Chicken embryo embryoblasts; BAC: Bacterial artificial chromosome; RFLPs: Restriction fragment length polymorphisms.

## Competing interests

The authors declare that they have no competing interests.

## Authors’ contributions

LC planned and carried out virus recombinant, selection and identification of recombinant BAC clone, and rescue of reconstructed viruses, prepared figures, and drafted the manuscript. BY, JH and WY performed animal experiment. TY performed characterization of viruses in cells. ZN carried out viral detection and sequence alignment. XD constructed transfer vector for homologous recombination. And CZ gave suggestion for this research and helped with overall planning and drafting of the manuscript. All authors read and approved the final manuscript.
